# Supplementary material for: Glycosylation of Epigallocatechin Gallate by Engineered Glycoside Hydrolases from Talaromyces amestolkiae: Potential Antiproliferative and Neuroprotective Effect of These Molecules
Source: Antioxidants (Basel). 2022 Jul 5;11(7):1325. doi: 10.3390/antiox11071325 (PMC9312355; doi:10.3390/antiox11071325)
Supplement: Supplementary file 1 [file antioxidants-11-01325-s001.zip › antioxidants-1764480-supplementary.pdf]

Suplementary material of the article:

Glycosylation of epigallocatechin gallate by engineered glycosyde hydrolases from *Talaromyces amestolkiae*: antiproliferative and neuroprotective properties of the novel glycosides.

AUTHORS LIST AND AFFILIATIONS:

Juan A. Méndez-Líter[1] (jmendez@cib.csic.es), Ana Pozo-Rodríguez[1] (ana.pozo@cib.csic.es), Enrique Madruga[2] (enrique.madruga@cib.csic.es), María Rubert[3] (mrubert@ucm.es), Andrés G. Santana[4] (andres.g.santana@csice.es), Laura Isabel de Eugenio[1] (lidem@cib.csic.es), Cristina Sánchez[3] (cristina.sanchez@quim.ucm.es), Ana Martínez[2] (ana.martinez@csic.es), Alicia Prieto[1] (aliprieto@cib.csic.es), and María Jesús Martínez[1*] (mjmartinez@cib.csic.es).

[1]Centro de Investigaciones Biológicas Margarita Salas, Department of Microbial and Plant Biotechnology, CSIC, Ramiro de Maeztu 9, 28040 Madrid, Spain.

[2]Centro de Investigaciones Biológicas Margarita Salas, Department of Structural and Chemical Biology, CSIC, Ramiro de Maeztu 9, 28040 Madrid, Spain.

[3]Universidad Complutense de Madrid, School of Biology, Department of Biochemistry and Molecular Biology, C/ de José Antonio Nováis 12, and Instituto de Investigación Hospital 12 de Octubre, 28040 Madrid, Spain.

[4] Instituto de Química Orgánica General, Department of Bioorganic Chemistry, CSIC. C/ Juan de la Cierva 3, 28006 Madrid, Spain.

Supplementary Data S1. Equations for maximum production and maximum conversion for EGCG glycosides

EGCG glucoside maximum production was adjusted to the following quadratic model equation: Production (g/L) = – 3.14841 + 0.005127 * Time + 0.151687 * [EGCG] + 0.099441 * [GF] + 2.72465 * [BGL-1-E521G].

EGCG glucoside maximum conversion was adjusted to the following quadratic model equation: Yield (%) = 16.89094 + 0.015827 * Time – 0.246401 * [EGCG] + 0.243565 * [GF] + 12.52816 * [BGL-1-E521G].

EGCG sophoroside maximum production was adjusted to the following quadratic model equation: Production (g/L) = 0.631575535 – 0.000879907 * Time + 0.024362149 * [EGCG] – 0.025933534 * [GF] – 0.57690485 * [BGL-1-E521G] – 5.72538E-06 * Time * [EGCG] + 7.38884E-05 * Time * [GF] + 0.002949491 * Time * [BGL-1-E521G] – 0.000594511 * [EGCG] * [GF] – 0.040613467 * [EGCG] * [BGL-1-E521G] + 0.095248319 * [GF] * [BGL-1-E521G].

EGCG sophoroside maximum conversion was adjusted to the following quadratic model equation: Yield (%) = – 18.50356536 + 0.038519128 * Time – 0.287082415 * [EGCG] + 0.574977698 * [GF] + 58.31594217 * [BGL-1-E521G] – 0.001449099 Time * [EGCG] + 0.00028466 * Time * [GF] + 0.012664991 * Time * [BGL-1-E521G] – 0.01379129 [EGCG] * [GF] – 1.404943926 * [EGCG] * [BGL-1-E521G] + 0.311310773 * [GF] * [BGL-1-E521G] + 1.21476E-05 * $Time^2$ + 0.021742508 $[EGCG]^2$ – 0.004837327 * $[GF]^2$ – 13.93947116 * $[BGL\text{-}1\text{-}E521G]^2$.

EGCG xyloside maximum production was adjusted to the following quadratic model equation: Production (g/L) = – 27.0092 + 0.107516 * Time + 0.352642 * [EGCG] – 0.121914 * [pNPX] + 11.10301 * [BxTW1-E495A] + 3.52688 * pH + 0.000064 * Time

* [EGCG] + 0.000268 * Time * [pNPX] + 0.000036 * Time * [BxTW1-E495A] – 0.019671 * Time * pH – 0.000032 * [EGCG] * [pNPX] + 0.021935 * [EGCG] * [BxTW1-E495A] + 0.000379 * [EGCG] *pH + 0.049546 * [pNPX] * [BxTW1-E495A] + 0.018371 [pNPX] * pH – 1.82195 * [BxTW1-E495A] + 7.24E-06 * Time^2 – 0.006808 * [EGCG]^2 – 0.000125 * [pNPX]^2 – 0.30183 * [BxTW1-E495A]^2 + 0.112643 pH^2.

EGCG xyloside maximum conversion was adjusted to the following quadratic model equation: Yield (%) = – 182.3495071 + 0.254393427 * Time + 3.57259018 * [EGCG] – 0.610354702 * [pNPX] + 30.19055765 * [BxTW1-E495A] + 40.24645123 * pH + 0.001349786 * Time * [EGCG] + 0.004772928 * Time * [pNPX] + 0.002106576 * Time * [BxTW1-E495A] – 0.07393721 * Time * pH – 0.02061489 * [EGCG] * [pNPX] + 0.289477271 * [EGCG] * [BxTW1-E495A] – 0.711293945 * [EGCG] * pH + 0.201917046 * [pNPX] * [BxTW1-E495A] + 0.072974668 * [pNPX] * pH – 6.774289987 * [BxTW1-E495A] * pH.
